# The Effects of Temperature on the Growth of a Lead-Free Perovskite-Like (CH_3_NH_3_)_3_Sb_2_Br_9_ Single Crystal for An MSM Photodetector Application

**DOI:** 10.3390/s21134475

**Published:** 2021-06-30

**Authors:** Chien-Min Hun, Ching-Ho Tien, Kuan-Lin Lee, Hong-Ye Lai, Lung-Chien Chen

**Affiliations:** Department of Electro-Optical Engineering, National Taipei University of Technology, Taipei 10608, Taiwan; dreamsmin@gmail.com (C.-M.H.); chtien@mail.ntut.edu.tw (C.-H.T.); t102658016@gmail.com (K.-L.L.); joe80314@gmail.com (H.-Y.L.)

**Keywords:** lead-free perovskite, MA_3_Sb_2_Br_9_, perovskite single crystal, perovskite photodetector

## Abstract

We have fabricated a photodetector based on (CH_3_NH_3_)_3_Sb_2_Br_9_ (MA_3_Sb_2_Br_9_) lead-free perovskite-like single crystal, which plays an important role in the optoelectronic characteristics of the photodetector as a perovskite-like photosensitive layer. Here, MA_3_Sb_2_Br_9_ single crystals were synthesized by an inverse temperature crystallization process with a precursor solution at three different growth temperatures, 60 °C, 80 °C, and 100 °C. As a result, a MA_3_Sb_2_Br_9_ single crystal with an optimum growth temperature of 60 °C presented a low trap density of 2.63 × 10^11^ cm^−3^, a high charge carrier mobility of 0.75 cm^2^ V^−1^ s^−1^, and excellent crystal structure and optical absorption properties. This MA_3_Sb_2_Br_9_ perovskite-like photodetector displayed a low dark current of 8.09 × 10^−9^ A, high responsivity of 0.113 A W^−1^, and high detectivity of 4.32 × 10^11^ Jones.

## 1. Introduction

Organic–inorganic lead halide composite perovskite materials have received widespread attention because of their excellent photoelectric properties. Due to the advantages of low exciton binding energy, high carrier mobility, long carrier diffusion lengths, wide light absorption range, high light absorption, and tunable optical band gap suitable for solar spectrum, they have quickly attracted widespread attention in the field of high-efficiency photovoltaic cells [1,2,3,4,5]. In recent years, the power conversion efficiency (PCE) of photovoltaic cells made of Pb-based organic–inorganic composite perovskite materials has increased from less than 5% to more than 25.5% [6,7,8,9]. At the same time, they have shown great application potential in the fields of photodetectors [10,11] and light-emitting diodes (LEDs) [12,13], but the above application research was mostly limited to polycrystalline thin film materials; there were few reports on the preparation and application of single-crystal materials. In the application of semiconductor materials, single-crystal materials are of great importance. There are a large number of grain boundaries and more defects in polycrystalline materials, so their physical properties are generally not as good as those of single-crystal materials. Optoelectronic devices made of polycrystalline materials, such as photovoltaic cells, generally have lower performance than single-crystal material devices. Therefore, the preparation of single-crystal perovskite materials and the study of their properties and applications have become important in this field [5,14,15,16,17,18].

However, although Pb-based perovskite solar cells have achieved high PCE, the toxicity of heavy metal Pb has a greater impact on human health and the environment. Therefore, the post-processing Pb content has to be considered [19,20]. Although the Pb content per square meter in perovskite solar panels was only a few hundred milligrams, this issue should be given attention when using this material on a large scale. To solve this problem, researchers have turned their attention to many low-toxic or lead-free perovskite materials. The development of lead-free organic–inorganic-composite perovskite materials is one of the most important research topics at present and also a challenging problem. So far, tin (Sn) [21,22], germanium (Ge) [23,24], copper (Cu) [25,26], bismuth (Bi) [27,28], and antimony (Sb) [29,30] have been used as replacements for the Pb in Pb-based perovskite materials. Sn was the earliest metal element that was considered to be a more environmentally friendly element with the potential to replace Pb. However, Sn^2+^ and Ge^2+^ were more unstable than Pb^2+^ in the air; they were easily and quickly oxidized into the more stable Sn^4+^ and Ge^4+^ forms in an oxidizing atmosphere, which led to rapid destruction of the perovskite structure [31,32,33,34]. It is important to solve the stability of Sn-based or Ge-based organic–inorganic-composite perovskite materials in the air, and carry out research on the growth and basic performance of new narrow-bandgap lead-free perovskite materials, which is the key to extending the application of perovskite materials in the optoelectronics field.

Perovskite is a promising material for applications of fast and tunable broadband and narrowband photodetectors [35]. Recently, a material with the same structure as perovskite or a similar one has appeared, A_3_Bi_2_X_9_ (A = MA or Cs; X = I, Br, and Cl), a bismuth halide material, which is a more typical perovskite-like material. Since Bi is a low-toxic element adjacent to Pb, A_3_Bi_2_X_9_ becomes a potential new light-absorbing material to replace Pb-based perovskite materials, which have better stability than MAPbI_3_ perovskites [36]. As an element of the same main group of Bi, Sb has an outer electron arrangement similar to that of Bi and has become a substitute material for Pb [37]. Han et al. reported that a photodetector based on MA_3_Sb_2_I_9_ microcrystals has a high responsivity of 40 A W^−1^ under monochromatic light (460 nm) [38]. Pan et al. reported a highly stable and lead-free Cs_3_Bi_2_I_9_ perovskite nanoplate for visible light photodetection applications, showing a maximum light responsivity of 33.1 mA W^−1^ under the irradiation of a 450-nm laser [26]. Zhai et al. synthesized submillimeter-size monocrystalline lead-free Cs_3_Sb_2_Br_9_ perovskite nanoflakes using an inverse temperature crystallization (ITC) method, demonstrating that the Cs_3_Sb_2_Br_9_ perovskite nanoflake photodetector has a fast response speed of 24/48 ms, a high responsivity of 3.8 A W^−1^, and an excellent detectivity of 2.6 × 10^12^ Jones [39]. However, the nano/microscale device size is too small and the nanoflakes were deposited on the electrodes at random, which limits the applications in the future. The responsiveness of lead-free perovskite photodetectors reported so far was limited, and the performance of the device was far below the requirements of sensitive photoelectric detection. Therefore, it is urgent to study this issue to develop high-performance, lead-free perovskite photodetectors.

Herein, we successfully prepared millimeter-sized, single-crystal, hexagonal, lead-free MA_3_Sb_2_Br_9_ perovskite via the ITC method. By adjusting the growth temperature and the constant temperature control of the oven, the morphology of the lead-free crystal can be controlled. Moreover, it was revealed that the high quality, smooth surface, and uniform geometric shape enabled the as-prepared crystals to have good optical properties and light absorption function, and allowed for the study of its MA_3_Sb_2_Br_9_ perovskite single crystal in metal–semiconductor–metal (MSM) photodetector applications.

## 2. Materials and Methods

### 2.1. Materials

Methylammonium bromide (MABr, 99.9%) was purchased from Lumtec (New Taipei, Taiwan). Antimony (III) bromide (SbBr_3_, 99.995%) was purchased from Alfa Aesar (Haverhill, MA, USA). Gamma-butyrolactone (GBL, ≥99.9%) was purchased from Echo Chemical (Miaoli, Taiwan). C_60_ Fullerene (C_60_, 99.95%) was purchased from Sigma-Aldrich (St. Louis, MO, USA). Silver slug (Ag, 99.999%) was purchased from Gredmann (Taipei, Taiwan).

### 2.2. Preparation of MA_3_Sb_2_Br_9_ Single Crystals and Photodetector Device

**Figure 1:**
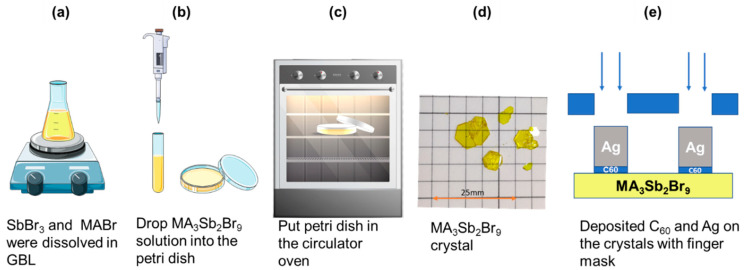
MA_3_Sb_2_Br_9_ single crystals’ growth process and a schematic diagram of an MSM-structured photodetector.

**Figure 2:**
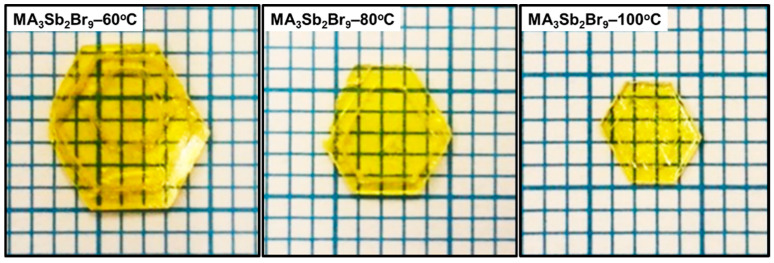
Photographs of MA_3_Sb_2_Br_9_ single crystals with different growth temperatures.

For the ITC process in Figure 1, MABr and SbBr_3_ precursors were dissolved at a 1.5:1.5 molar ratio in 1 mL GBL solvent. The solution was stirred at 700 rpm for 30 min to ensure proper dissolution. After adding it to the Petri dish, the solution was maintained at 60 °C, 80 °C, or 100 °C until the solution was completely evaporated in the hot air circulation oven. During the heating process, the Petri dish had to be covered to prevent the solution from evaporating too quickly. For a while, a few small MA_3_Sb_2_Br_9_ crystallites appeared in the liquid. The MA_3_Sb_2_Br_9_ single crystals gradually grew into a hexagonal shape and became larger in solution. Moreover, the growth times of the final MA_3_Sb_2_Br_9_ single crystals at 60 °C, 80 °C, and 100 °C were about 120, 72, and 36 h, respectively. As a result, the crystal dimensions (7 × 8 × 4 mm), (5 × 6 × 3.5 mm), (4 × 5 × 3 mm) of the MA_3_Sb_2_Br_9_ single crystals were obtained at 60 °C, 80 °C, and 100 °C, respectively (Figure 2). The lower growth temperature makes the precursor solution volatilize more slowly, which promotes a low growth rate to produce larger and better single crystals. Conversely, the final crystal grown at high temperature becomes smaller and has more defects due to the fast growth rate and faster volatilization of the precursor solution. Finally, 20 nm C_60_ and 300 nm Ag were deposited on the MA_3_Sb_2_Br_9_ single crystals with a finger mask to give the MSM photodetector (Ag/C_60_/MA_3_Sb_2_Br_9_/C_60_/Ag) an effective illuminated area of 3.84 × 10^−2^ cm^2^.

### 2.3. Characterization

Morphologies of the MA_3_Sb_2_Br_9_ single crystals were inspected using a field-emission scanning electron microscope (FESEM, ZEISS Sigma, ZEISS, Munich, Germany). Crystal crystallization qualities were characterized using an X-ray diffractometer (X’Pert PRO MRD, PANalytical, Almelo, Netherlands). The optical properties were measured by a fluorescence spectrophotometer (F-7000, Hitachi, Tokyo, Japan) and a UV–VIS/NIR spectrophotometer (UH-4150, Hitachi). The electrical characteristics of the devices were measured using a Keithley 2420 sourcemeter under dark and 100 mW cm^−2^ illumination conditions. The responsivity characteristics of the MSM photodetectors were collected using a Keithley 2420 sourcemeter to record the photocurrent at a bias voltage of 20 V under the 140 W Xe lamp for illumination. The incident optical power was measured by laser power and energy meters (NOVAII 7Z01550, Ophir Optronics, Jerusalem, Israel). All the characterizations were measured at room temperature.

## 3. Results and Discussion

**Figure 3:**
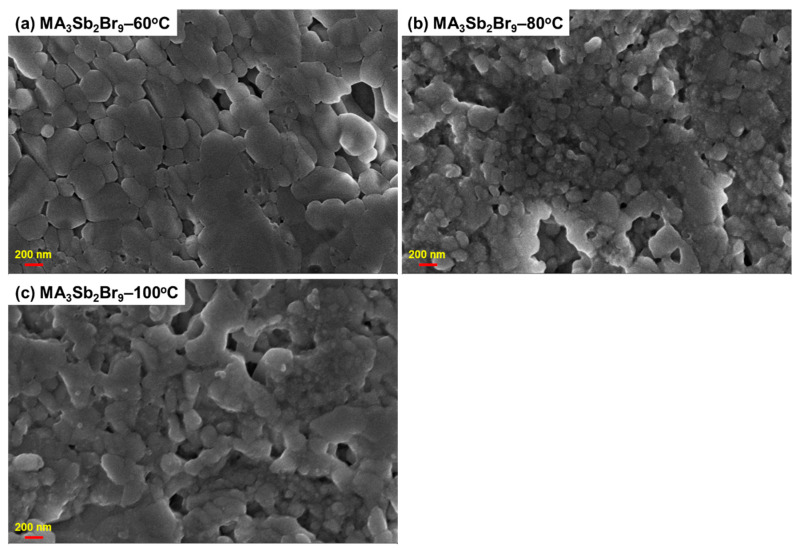
Field-emission scanning electron microscope (FESEM) micrographs of MA_3_Sb_2_Br_9_ single crystals with different growth temperatures.

Figure 3 shows the FESEM surface morphologies of the MA_3_Sb_2_Br_9_ single crystals with different growth temperatures. It can be seen that, when the growth temperature of the perovskite crystal was 60 °C, a dense and crack-free morphology with a large grain size could be obtained. On the other hand, when the growth temperatures of the perovskite crystals were 80 °C and 100 °C, relatively irregular grain size and pinholes were observed on the perovskite crystal. This is because the pinholes or cracks act as nonradiative recombination centers, which could cause poor device performance. Therefore, the optimized growth temperature helps to create high-crystallinity crystals without pinholes and cracks, which can promote the separation and transport process of photo-excited carriers.

**Figure 4:**
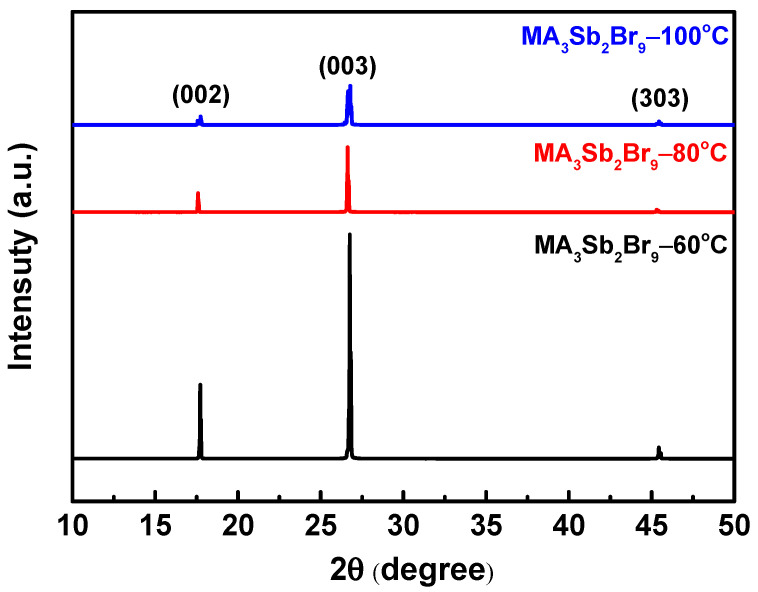
X-ray diffraction (XRD) patterns of MA_3_Sb_2_Br_9_ single crystals with different growth temperatures.

To further understand the situation of a MA_3_Sb_2_Br_9_ single crystal, an X-ray diffraction analysis was carried out; the result is shown in Figure 4. In the range of 10 to 50°, the MA_3_Sb_2_Br_9_ single crystal synthesized under the three growth temperature conditions has a good overlap of diffraction peaks, which proves that their composition was consistent. Moreover, four main peaks appeared at the diffracted peak positions 2θ = 17.73°, 26.76°, 45.32°, and 55.24°, corresponding to the crystal planes (002), (003), (303), and (042), respectively. Comparing with the crystal data in the literature [38,40], it was found that these were the main characteristic peaks of MA_3_Sb_2_Br_9_ single crystal, which confirmed the formation of trigonal P3-m1 symmetry MA_3_Sb_2_Br_9_ single crystal, and the corresponding lattice parameters a = 7.6918 Å and c = 7.5842 Å. From the three sets of diffraction peak intensities, it could be seen that the main peak of MA_3_Sb_2_Br_9_ single crystal grown at 60 °C was sharp and the full width at half maximums (FWHMs) (0.1053°) was very small, which proves that the crystallinity was very high. Compared with the other two growth temperatures, the crystal quality of the single crystal grown at 100 °C was the worst, and the degree of crystallization was not high. The quality of the single crystal grown at 80 °C was slightly better than that of the one grown at 100 °C.

**Figure 5:**
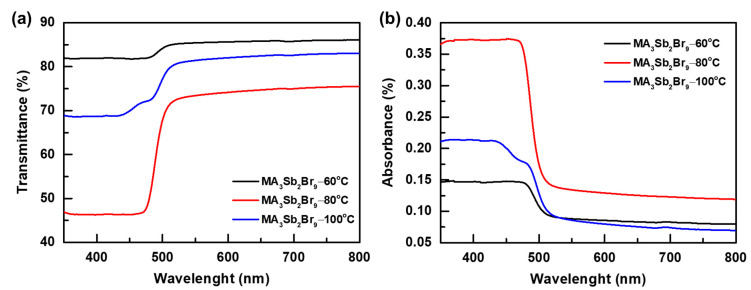
(**a**) Transmittance and (**b**) absorbance spectra of MA_3_Sb_2_Br_9_ single crystals with different growth temperatures.

Figure 5a shows the transmittance spectra of MA_3_Sb_2_Br_9_ single crystals at different growth temperatures. MA_3_Sb_2_Br_9_ single crystals grown at different temperatures of 60 °C, 80 °C, and 100 °C show transmittances of 74.3%, 82.1%, and 85.7% at wavelengths higher than 500 nm, respectively. In addition, the sharp drop in transmittance observed below 500 nm was due to the strong absorption of the MA_3_Sb_2_Br_9_ single crystal. Such a strong absorption undoubtedly promotes the generation of effective exciton within the material. Figure 5b shows the absorption spectra of MA_3_Sb_2_Br_9_ single crystals obtained at different growth temperatures. It can be seen that the MA_3_Sb_2_Br_9_ single crystal synthesized at different growth temperatures of 60 °C, 80 °C, and 100 °C exhibited different absorption levels in the visible light range. Among them, the absorbance of the MA_3_Sb_2_Br_9_ single crystal was the highest at the growth temperature of 60 °C, followed by 80 °C, and lowest at 100 °C. This shows that the MA_3_Sb_2_Br_9_ single crystal synthesized at a growth temperature of 60 °C not only has high crystal quality, but also has the best light absorption. The MA_3_Sb_2_Br_9_ single crystal under 80 °C has better light absorption than that of 100 °C. The band gap of these crystals varied from 2.38 to 2.48 eV and was calculated by a Tauc plot. The UV-Vis absorption of the MA_3_Sb_2_Br_9_ single crystal obtained under the conditions of the three combinations was consistent with the results of the previous SEM and XRD characterization.

**Figure 6:**
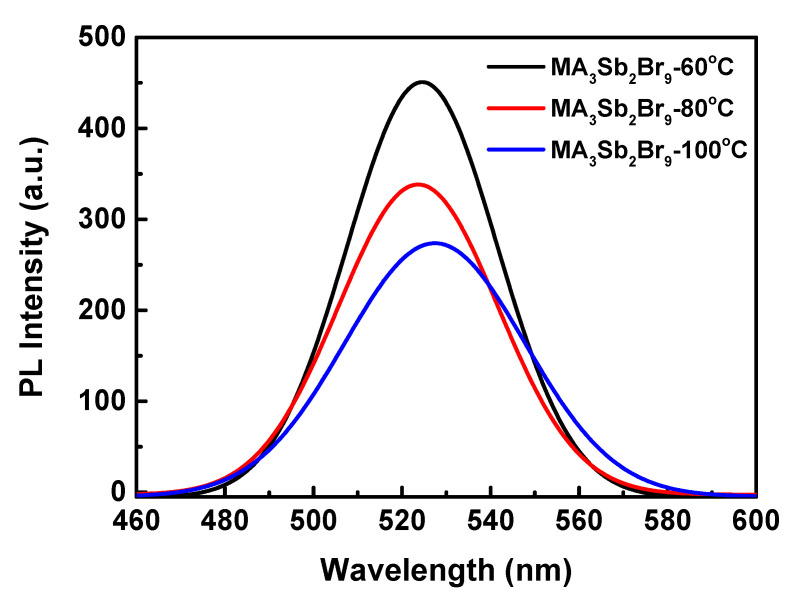
Photoluminescence (PL) spectra of MA_3_Sb_2_Br_9_ single crystals with different growth temperatures.

After discussing the light absorption properties of MA_3_Sb_2_Br_9_ single crystal with different growth temperatures, its photoluminescence (PL) properties were studied, and the PL spectra were obtained, as shown in Figure 6. It can be seen that the three kinds of MA_3_Sb_2_Br_9_ single crystals with different growth temperatures had a very narrow PL emission peak at the wavelength of 523–527 nm, and FWHMs of 39.9, 42.4, and 47.9 nm. The PL intensities of single crystals at the three growth temperatures were different. The MA_3_Sb_2_Br_9_ single crystal grown at 60 °C had the highest PL intensity, followed by 80 °C, and 100 °C was the lowest. This was consistent with the conclusion drawn from the previously analyzed absorbance spectra. For a perovskite material, the narrow PL emission peak may also be caused by the characteristics of the sharp absorption edge, according to Figure 5, except for the crystal quality [41]. It was proven that the MA_3_Sb_2_Br_9_ single crystal with good light absorption performance also had the best PL performance. In addition, the MA_3_Sb_2_Br_9_ single crystal grown at 60 °C corresponded to the narrow PL peak that appeared on both sides (highly symmetrical), indicating that the single crystal MA_3_Sb_2_Br_9_ had a low density of defect states at this growth temperature. This was consistent with the previously proved MA_3_Sb_2_Br_9_ single crystal quality and the best crystallization results.

**Figure 7:**
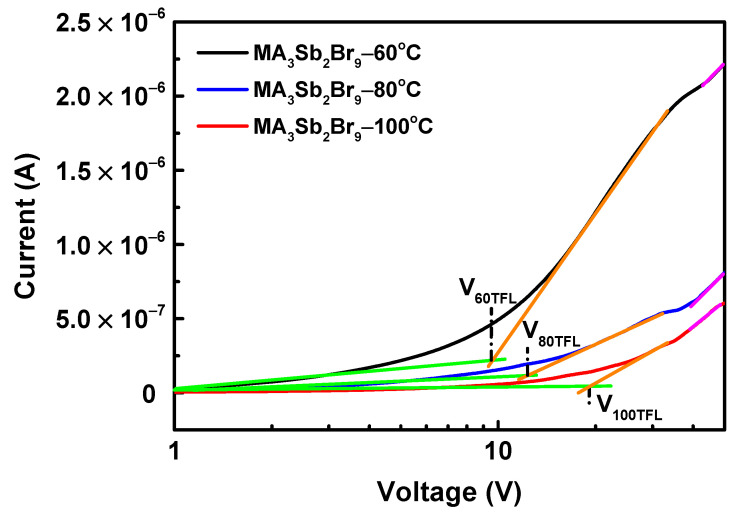
Dark current–voltage (*I–V*) curves of MA_3_Sb_2_Br_9_ single-crystal photodetector with different growth temperatures.

To determine the charge transport and trap-state density of single crystals, the dark current–voltage (*I–V*) curve of the MA_3_Sb_2_Br_9_ single crystal photodetector device was measured by a space-charge-limited current (SCLC) analysis. The defects of perovskite will trap carriers, thereby reducing the concentration of free carriers. Under a lower bias, the current and voltage are linearly related. The injected carriers are continuously captured by defects when the voltage gradually increases. When the defect is filled, the current will increase nonlinearly—that is, a kink-point appears on the *I–V* curve. The voltage corresponding to this point is the trap-filled limit voltage (*V_TFL_*), which can be obtained from the intersection of the tangent of the linear growth part and the tangent of the nonlinear growth part. Figure 7 presents the dark *I–V* curve at different growth temperatures. Under a lower bias voltage, it was a linear Ohmic region (green line); the high bias region was the trap-filling region (orange line), and the transition region from Ohmic to Child’s law was the trap-filling limit region (purple line). The trap-state density could be calculated by Equation (1), as follows [14]:

(1) $$n_{\mathrm{trap}}=\frac{2\varepsilon_{0}\varepsilon_{r}V_{TFL}}{qL^{2}},$$

where *q* is the elementary charge; n_trap_ is the trap-state density; *L* is the single crystal thickness; and *ε_r_* and *ε*_0_ represent the elative dielectric constant and vacuum permittivity, respectively. After fitting, the corresponding *V_TFLs_* of the MA_3_Sb_2_Br_9_ single crystals at growth temperatures of 60 °C, 80 °C, and 100 °C were 9.5, 12.3, and 19.1 V, respectively. The trap-state densities can be calculated as 2.63 × 10^11^, 4.44 × 10^11^, and 9.40 × 10^11^ cm^−3^, respectively, indicating that the lower trap-state density of the perovskite MA_3_Sb_2_Br_9_ crystal grown at 60 °C led to better crystalline quality and higher carrier mobility, which are vital to improve the charge transport of an optoelectronic device. In addition, the charge carrier mobility of MA_3_Sb_2_Br_9_ was also estimated according to the Mott–Gurney law [14]:

(2) $$J=\frac{9}{8}\varepsilon_{0}\varepsilon_{r}\mu \frac{V^{2}}{L^{3}},$$

where *J*, *ε*_0_, *ε_r_*, *μ*, *V*, and *L* are the dark current, the relative dielectric constant, the vacuum permittivity, the charge carrier mobility of single crystals, the applied voltage, and the single crystal thickness, respectively. As a result, the calculated charge carrier mobility of the MA_3_Sb_2_Br_9_ single crystals at growth temperatures of 60 °C, 80 °C, and 100 °C was 0.75, 0.18, and 0.09 cm^2^ V^−1^ s^−1^, respectively. In particular, the MA_3_Sb_2_Br_9_ single crystal grown at 60 °C was larger than the previously reported value (0.4 cm^2^ V^−1^ s^−1^) [37], which indicates a predictable improvement in the photocurrent of the photodetector.

**Figure 8:**
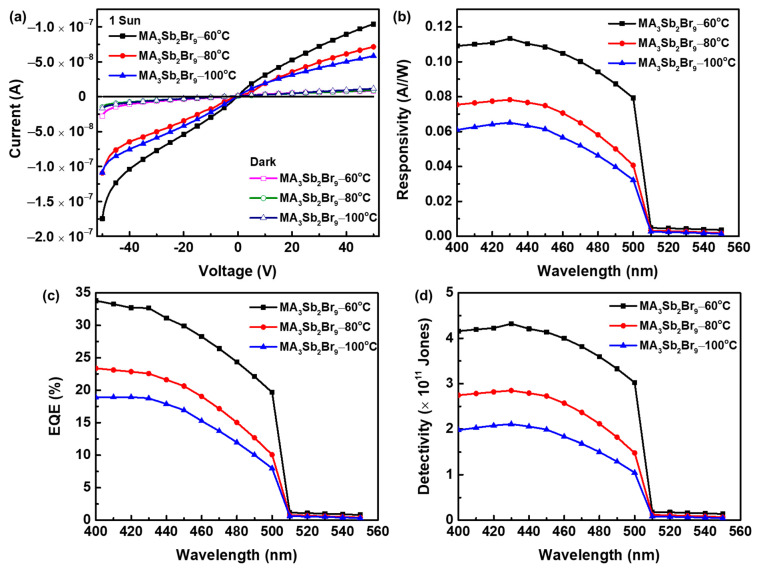
(**a**) Current–voltage (*I–V*) curves of MA_3_Sb_2_Br_9_ single-crystal photodetectors with different growth temperatures under dark and 1 sun illumination. (**b**–**d**) Wavelength-dependent responsivity, external quantum efficiency, and detectivity of MA_3_Sb_2_Br_9_ single-crystal photodetectors with different growth temperatures under 20 V.

Figure 8a depicts the *I–V* curve of MA_3_Sb_2_Br_9_ single-crystal photodetectors with different growth temperatures under dark and 1 sun illumination. The photocurrents of the MA_3_Sb_2_Br_9_ single crystals at growth temperatures of 60 °C, 80 °C, and 100 °C at 0 V bias were 1.18 × 10^−9^, 1.15 × 10^−9^, and 1.04 × 10^−9^ A, respectively. Compared with those at 80 °C (8.95 × 10^−9^ and 7.15 × 10^−8^ A) and 100 °C (1.15 × 10^−8^ and 5.85 × 10^−8^ A) of the MA_3_Sb_2_Br_9_ single-crystal photodetector, the dark current and photocurrent of the MA_3_Sb_2_Br_9_ single-crystal photodetector grown at 60 °C were 8.09 × 10^−9^ and 1.04 × 10^−7^ A (@50 V), respectively. The relatively low dark current could be attributed to the high-quality MA_3_Sb_2_Br_9_ single crystal. Moreover, when the bias voltage was 20 V, the light/dark ratios of the MA_3_Sb_2_Br_9_ single crystals at growth temperatures of 60 °C, 80 °C, and 100 °C were 10.2, 7.6, and 6.3, respectively. Responsivity (R), external quantum efficiency (EQE), and detectivity (D*) were important performance indicators of photodetectors. The wavelength-dependent responsivity, EQE, and detectivity of MA_3_Sb_2_Br_9_ single-crystal photodetectors with different growth temperatures are presented in Figure 8b–d, measured at a bias voltage of 20 V. R, EQE, and D* were defined as follows [14,37]:

(3) $$R=\frac{I_{ph}-I_{dark}}{P_{in}},$$

(4) $$\mathrm{EQE}=R\frac{hc}{e\lambda },$$

(5) $$D*=\frac{R}{\sqrt{2eI_{dark}/A}},$$

where *I_ph_* is the photocurrent, *I_dark_* is the dark current, *P_in_* is the illumination power, *e* is the electronic charge constant, *h* is the Planck’s constant, *c* is the light velocity, *λ* is the wavelength of the incident light source, and *A* represents the effective illumination area of the photodetector. We observed that the MA_3_Sb_2_Br_9_ single-crystal photodetector grown at 60 °C attained a maximum responsivity of 0.113 A W^−1^, EQE of 32.7%, and detectivity of 4.32 × 10^11^ Jones under monochromatic light with a wavelength of 430 nm, while 0.078 and 0.065 A W^−1^ responsivity, and 22.6% and 18.8% EQEs, as well as 2.85 × 10^11^ and 2.11 × 10^11^ Jones detectivities, were calculated for MA_3_Sb_2_Br_9_ single-crystal photodetectors grown at 80 °C and 100 °C, respectively. The increase in responsivity might be attributed to the lower trap-state density and the high optical absorption of the MA_3_Sb_2_Br_9_ single crystal grown at 60 °C, with the improved carrier injection helping to enhance the photocurrent of the photodetector. On the other hand, reducing the trap-state density could result in a slow recombination rate of the photodetector, which is beneficial in terms of the suppression of the dark current and enhanced detection capability. To compare Cs_3_Sb_2_Br_9_-based photodetector with a very narrow band width (480 ± 4 nm only). This work exhibits a wide responsivity and detectivity range, from 400 to 500 nm [42].

**Figure 9:**
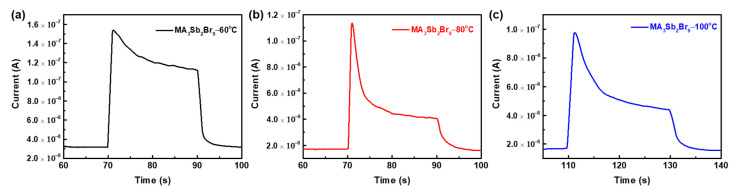
Photocurrent response of the MA_3_Sb_2_Br_9_ single-crystal photodetector with different growth temperatures of MA_3_Sb_2_Br_9_−60°C (**a**), MA_3_Sb_2_Br_9_−80°C (**b**), and MA_3_Sb_2_Br_9_−100°C (**c**) under light illumination of 100 mW cm^−^^2^ (bias @20 V).

Figure 9 shows the time-dependent photocurrent curve of the MA_3_Sb_2_Br_9_ single-crystal photodetector with different growth temperatures under a light intensity of 100 mW cm^−2^ and an applied bias of 20 V in order to measure the response time. The rise and fall times of the detector were defined as the time required for the photocurrent to increase from 10% to 90% and decrease from 90% to 10% of the steady-state saturation current, respectively. The rise and fall times of the MA_3_Sb_2_Br_9_ single-crystal photodetector grown at 60 °C were 47.1 and 1162 ms, which was faster than the rise times (58.1 and 59.5 ms) and fall times (3738 and 4230 ms) of the MA_3_Sb_2_Br_9_ single-crystal photodetectors grown at 80 °C and 100 °C, respectively, indicating that single crystal grown at the lower temperature of 60 °C improves the separation efficiency of photoinduced charge carriers.

**Figure 10:**
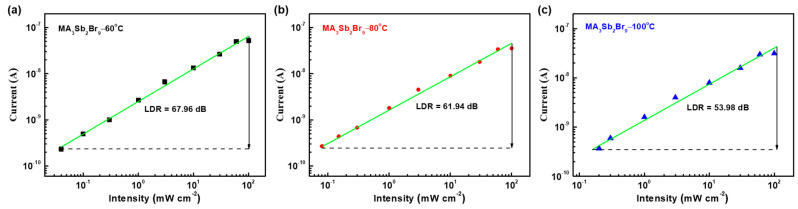
Illumination power density-dependent photocurrent of the MA_3_Sb_2_Br_9_ single-crystal photodetector with different growth temperatures of MA_3_Sb_2_Br_9_−60 °C (**a**), MA_3_Sb_2_Br_9_−80 °C (**b**), and MA_3_Sb_2_Br_9_−100 °C (**c**) at 20 V bias (430 nm).

We characterized the linear dynamic range (LDR) of the prepared MA_3_Sb_2_Br_9_ single-crystal photodetector. A plot of the photocurrent versus the illumination power density at 430 nm (bias @20 V) for the MA_3_Sb_2_Br_9_ single-crystal photodetectors is displayed in Figure 10a–c. The minimum detectable light intensities were 4 × 10^−5^, 8 × 10^−5^, and 2 × 10^−4^ W cm^−2^ for the MA_3_Sb_2_Br_9_ single crystals at growth temperatures of 60 °C, 80 °C, and 100 °C, respectively. Here, LDR was obtained by LDR = 20LOG(*I_upper_*/*I_lower_*), where *I_upper_* and *I_lower_* represent the upper and lower detectable currents, respectively. Therefore, the LDRs of the MA_3_Sb_2_Br_9_ single-crystal photodetectors were 67.96, 61.94, and 53.98 dB, respectively. A comparison of the lead-free Sb-based perovskite photodetectors considered in other works is given in Table 1.

## 4. Conclusions

We have successfully fabricated lead-free perovskite-like MA_3_Sb_2_Br_9_ single crystals using an ITC method. Compared with the other crystal growth temperatures, the obtained MA_3_Sb_2_Br_9_ single crystal at 60 °C presented a dense and crack-free morphology with large grain size, excellent crystallinity, better charge carrier mobility (0.75 cm^2^ V^−1^ s^−1^), and lower trap state density (2.63 × 10^11^ cm^−3^). Moreover, the MA_3_Sb_2_Br_9_ single-crystal photodetector showed a lower dark current (8.09 × 10^−9^ A), high responsivity (0.113 A W^−1^), and high detectivity (4.32 × 10^11^ Jones). In addition, the perovskite single crystal had extremely low defect density and few interface defects, which was conducive to the design of more stable optoelectronic devices. Lead-free Sb-based perovskite materials have a similar band gap as Pb-based perovskite materials (~2.2 eV). The operational temperature and water sensitivity should be investigated in the future.

## Figures and Tables

**Table 1:** Comparison of lead-free Sb-based perovskite photodetectors.

Device Structure	Preparation Method	Rise /Decay Times (ms)	Responsivity (A/W)	EQE (%)	Detectivity (Jones)	Ref.
ITO/MA_3_Sb_2_I_9_ single crystals/ITO	Slowly cooling process	0.4/0.9	40	-	10^12^	[39]
ITO/MA_3_Sb_2_Br_9_ single crystals/ITO	Slowly cooling process	1000	0.03	-	5 × 10^8^	[39]
Au/Ti/Si/SiO_2_/Cs_3_Sb_2_Br_9_ nanoflakes/Ti/Au	ITC process	24/48	3.8	-	2.6 × 10^12^	[40]
Au/Si/SiO_2_/Cs_3_Sb_2_Br_9_ single crystals/Au	Solvothermal process	0.2/3.0	2.29	18	3.77 × 10^12^	[42]
Ag/C_60_/MA_3_Sb_2_Br_9_ single crystals/C_60_/Ag	ITC process (oven)	47.1/1162	0.113	32.7	4.32 × 10^11^	This work

## Data Availability

Not applicable.
